# Burnout Syndrome and Sexual Disorders Among Vietnamese Female Nurses and Midwives at Tu Du Hospital: A Frontline Hospital-Based Cross-Sectional Study

**DOI:** 10.1089/whr.2024.0193

**Published:** 2025-07-02

**Authors:** Thanh Hai Pham, Minh Tuan Vo, Phuc Nhon Nguyen

**Affiliations:** ^1^ Tu Du Clinical Research Unit (TD-CRU), Tu Du Hospital, Ho Chi Minh City, Vietnam.; ^2^ Department of Obstetrics and Gynecology, University of Medicine and Pharmacy at Ho Chi Minh City, Ho Chi Minh City, Vietnam.; ^3^ Department of Pregnancy Pathology, Tu Du Hospital, Ho Chi Minh City, Vietnam.

**Keywords:** midwives, nurses, burnout syndrome, sexual dysfunction, Vietnam, women health

## Abstract

**Background:**

Burnout syndrome has become a great concern worldwide in recent decades. It can also lead to a negative effect on quality of work, life, and sexual dysfunction. This study assesses the prevalence of burnout syndrome and its association with sexual dysfunction among female nurses and midwives at a frontline hospital.

**Material and Methods:**

This cross-sectional study was conducted at Tu Du Hospital in Vietnam between February 1, 2023 and March 31, 2023. The study enrolled 485 female nurses and midwives. Among them, 426 women were eligible for the assessment of sexual disorders. This study used the Maslach Burnout Inventory–Human Services Survey toolkit to investigate burnout syndrome and the Female Sexual Function Index (FSFI) to determine sexual dysfunction.

**Results:**

Of the 485 respondents, the burnout rate was 27.0%, of which emotional exhaustion (EE), depersonalization, and diminished personal achievement (PA) were found at 13.2%, 6.0%, and 18.4%, respectively. Factors relating to burnout included young age, work duration of less than 10 years, sleep disorders, high workload, insufficient salary, and job dissatisfaction. Among 426 respondents completing the FSFI tool, 12.0% of female health care workers experienced sexual dysfunction, of which decreased sexual desire appeared in 42.0% of participants. EE at work was associated with sexual health issues and almost all sexual dysfunction factors with a weak negative correlation coefficient. Reduced PA was related to general sexual status, anorgasmia, and dyspareunia with a weak positive correlation coefficient.

**Conclusion:**

Overall, the prevalence of burnout and sexual disorders were 27.0% and 12.0%, respectively. Importantly, work-related factors were the main factors associated with burnout syndrome. EE and reduced PA were related to sexual disorders. These results underscore the need to assess support for the professional well-being of nurses and midwives. Further national-database studies are required to strengthen these findings.

## Introduction

According to World Health Organization, burnout syndrome is a state of mental, emotional, and physical exhaustion resulting from overwhelming stress at the workplace that has not been successfully managed. In other words, burnout develops secondary to long-term occupational stress.^1^ Theoretically, the development of burnout according to the socio-cognitive theory of self-efficacy includes efficacy crisis, low personal realization, emotional exhaustion (EE), and cynicism/depersonalization (DP).^2^

Globally, the prevalence of burnout is variable depending on the occupation.^3–6^ Among people working in the field of health care, the rate ranged from 0% to 80.5%.^7,8^ In accordance with Hiver et al., physicians’ burnout prevalence rates ranged from 2.5% to 72.0%. Using the dimensions of the Maslach Burnout Inventory (MBI) tool including EE, DP, and reduced personal accomplishment (PA), three definitions of burnout syndrome can be considered: tri-, bi-, and unidimensional definitions. Therefore, the variability depends on the dimensional definition. According to a meta-analysis by Hiver et al., the pooled prevalence rate of burnout among European physicians was estimated at 7.7% with the tridimensional definition, 19.7% with the bidimensional definition, and 43.2% with the unidimensional definition.^9^

To date, burnout risk could commonly occur at any age and women reached more frequent burnout than men (80.27% vs. 19.73%).^10,11^ According to Miguel-Puga et al., compared with men, women reporting pre-existent anxiety may be more prone to acute stress in the same environment and working circumstances.^12^ This phenomenon affects seriously the quality of work and life.^13^

Importantly, chronic burnout could affect libido and subsequently disturb the sexual life.^14^ The prevalence of sexual dysfunction among physicians experiencing burnout (45.51%) was much higher than that observed in physicians without burnout (24.19%).^15^ Thus, this issue should be relevant to raise awareness of health care managers and to seek help for such problems, especially, in low-resource settings such as Vietnam.^16,17^ As previously mentioned, the burnout syndrome among midwives could impact negatively on themselves as well as the patients in their care.^18,19^

As far as we know, the impact of coronavirus disease (COVID-19) pandemic has increased dramatically the burnout syndrome among health care workers.^20^ Many medical personnel have left the health care system due to the stress of jobs.^21–23^ However, knowledge about the stressful issues among female nurses and midwives has not been fully reported. Tu Du Hospital is an 1800-bed tertiary referral hospital involving obstetric and gynecological field in the southern region of Vietnam. In general, the establishment managed about 700,000 outpatients and 82,125 hospitalized patients, a total of 43,500 births, and 37,569 surgeries. Almost all health care workers are women; thus, their quality of work and life is the most concerned. Through this study, we purposed to document the prevalence of burnout syndrome and female sexual disorders among female health care personnel at our maternity hospital. In addition, the study aimed to address the relationship between burnout syndrome and sociodemographic characteristics and job characteristics as well as between burnout and sexual disorders. To the best of our knowledge, this is the first study exploring the effect of burnout syndrome on sexual dysfunction among midwives and nurses in Vietnam.

## Materials and Methods

### Study population

The cross-sectional study was designed between February 1, 2023 and the end of March, 2023, at Tu Du Hospital in the southern Vietnam. Ethical approval was approved by the Ethics Council in Biomedical Research Decision at Ho Chi Minh City University of Medicine and Pharmacy with the number 763/HDDD-DHYD dated October 20, 2022. All personal information and diseases of participants are kept confidential, encrypted, and used only for scientific research purposes.

### Inclusion criteria

During this period, the study included the participants who voluntarily agreed to participate completed an anonymous survey using the Maslach Burnout Inventory–Human Services Survey (MBI–HSS) tool for assessing burnout syndrome. The female nurses and midwives who had sexual intercourse within 1 month were invited to fill in the survey form of the Female Sexual Function Index (FSFI) tool as an assessment instrument.

### Exclusion criteria

The health care workers diagnosed with psychiatric disorders and under medication were excluded from the study. In addition, the study also excluded the participants who had been treated for sexual disorders (applying Kegel exercises, hormone replacement therapy, and traditional medicament). All unsuitable responses for analysis due to insufficient information or identical responses concerning important variables were also excluded from the analysis.

### Sample size

To calculate the required sample size for this study, the sample size calculation formula for cross-sectional studies was calculated as follows: n = [Z^2^_(1-α_/_2)_ p(1-p)]/d.^2.24^

Considering a 95% confidence level and an estimation of type I error of 1%, the prevalence of work-related burnout in health care workers was 0.792.^25^ The minimum sample size was 438.

### Sample selection steps

#### Step 1

We selected staff for participation in the study from specialized units that organize care and treatment of patients.

#### Step 2: Selecting participants

We used random sampling to select research subjects from among those identified as midwives and nurses working at the selected units.

#### Step 3

**FIG. 1.**
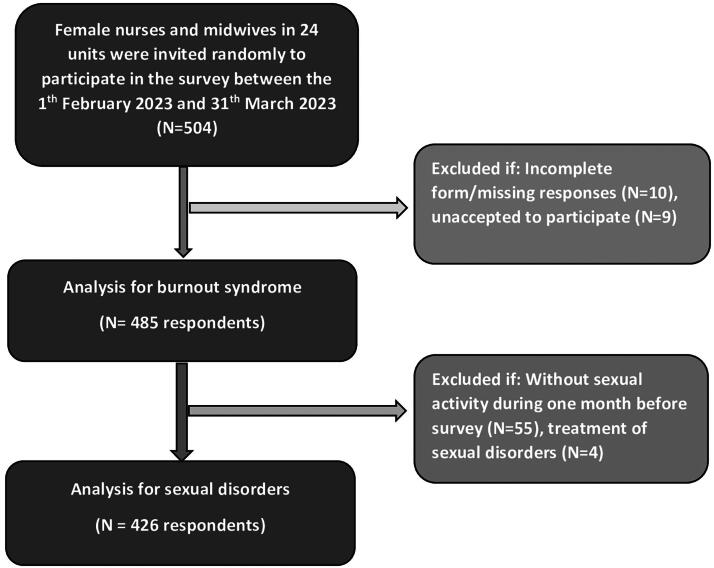
Study flowchart in the present study.

Determine the number of samples to be selected in each obstetrics and gynecology unit and recruitment (Supplementary Table S1). Corresponding to the estimated minimum sample size of 438, the study calculated the minimum estimated sample size for each unit (438 samples/24 units equals to18.25 female nurses and midwives/unit). Depending on the total of female nurses and midwives at each unit, approximately 20%–40% of female nurses and midwives were selected by stratified random sampling to participate in the study. The questionnaire distribution was given to 15–23 nurses and midwives/unit. In total, 504 questionnaire forms were sent. Among them, 485 samples were finally collected. Out of 485 female staffs, 426 nurses and midwives having sexual activity within 1 month eligible for inclusion criteria were invited to complete the FSFI form for investigating sexual dysfunction (Fig. 1).

#### Step 4: Data collection

At each unit, we set up a private sample collection room with a temperature of around 25°C and provided the most comfortable environment for the participants. First, we disseminated the meaning, objectives, and steps of the study. The sample collection team consisted of two trained midwives, each collecting two participants at two separate tables. One investigator collected the consent to participate in the study and 30 self-completed questions within 10 minutes. One investigator instructed on how to conduct the MBI–HSS survey (22 questions taking about 10 minutes) and FSFI (19 questions taking about 10 minutes) on Google Forms. It is recommended to complete the Google Form within 24 hours after being consulted. We periodically checked this implementation at 7 A.M. every day and reminded the participants using organization email. In case of the individual did not complete the survey within 7 days, the respondent would be considered as an excluded sample. Data were anonymized to ensure confidentiality.

### Study tools

#### MBI–HHS questionnaire

The original version of 22 items of the MBI–HSS questionnaire were used.^26^ This questionnaire is designed for health care workers and includes 22 items divided into 3 groups: EE with 9 questions;^1–3,6,8,13,14,16,20^ DP with 5 questions;^5,10,11,15,22^ PA with 8 questions.^4,7,9,12,17–19,21^

Each question was measured on a 7-point Likert scale from “never” (=0), “a few times a year or less” (=1), “one a month or less” (=2), “a few times a month” (=3), “once a week” (=4), “a few times a week” (=5), “every day” (=6).

The EE, DP, and PA were classified into low, medium, and high levels according to Maslach^26^ as below:

**Table:** 

Burnout	Emotional exhaustion	Depersonalization	Personal achievement
High	≥27	≥10	0–33
Medium	19–26	6–9	34–39
Low	0–18	0–5	≥40

Individuals with disorders in one of the three subgroups of EE, DP, or PA at a high level are considered to have burnout syndrome.

#### Prepare Vietnamese version of the MBI-HSS questionnaire

The Vietnamese version had been translated and revised by professional translators and an expert panel. Then, the translated version was compiled using Google Forms software. The survey form was distributed and collected anonymously.

Following translation, we agreed with the experts to conduct a pilot interview for 110 female nurses and midwives at Tu Du Hospital. The pilot interview was conducted in the same way as the sample collection process of the study to assess the reliability of the Vietnamese-translated version. Scale of the Vietnamese version of the MBI-HSS questionnaire as follows:

**Table:** 

Subscales	Number of questions	Cronbach’s alpha (*N* = 110)
Emotional exhaustion (EE)	9	0.95
Depersonalization (DP)	5	0.88
Personal achievement (PA)	8	0.87

The analysis results show that all aspects of the Vietnamese version of the MBI–HSS questionnaire have Cronbach’s alpha >0.7, achieving the accepted reliability coefficient. This translation was easy to understand by all participants. The pilot interviews were not included in the research sample. No changes were made to the translated version after the pilot study.

#### Sexual dysfunction questionnaire (FSFI)^27^

Decreased desire: Total score of decreased desire = question 1 + question 2; has 2 values “yes” and “no”; “yes” if the decreased desire score <4.28.

Decreased excitement: Total score of decreased excitement = question 3 + question 4 + question 5 + question 6; has 2 values “yes” and “no”; “yes” if the decreased excitement score <5.08.

Lack of vaginal lubrication: Total score of lack of vaginal lubrication = question 7 + question 8 + question 9 + question 10; has 2 values “yes” and “no”; “yes” if the lack of vaginal lubrication score <5.45.

Difficulty achieving orgasm: Total score of difficulty achieving orgasm = question 11 + question 12 + question 13; has 2 values of “yes” and “no”; “yes” if the difficulty in achieving orgasm score is <5.05.

### Data processing

Data were analyzed using the R statistical package version 4.2.2 (R foundation for statistical computing, Vienna, Austria; version 4.2.2).

We described quantitative variables as mean and standard deviation (SD) if normally distributed, by median, and quartile if non-normally distributed. The normality of the scales was evaluated by the Kolmogorov–Smirnov test and nonparametric and parametric tests were accordingly selected for further analysis.

Quantitative variables consist of: age (year), duration of work (year), monthly income (VND), the mean score of EE, DP, and PA in the MBI–HSS tool, and the mean score of each item in the FSFI tool.

We described qualitative variables as absolute numbers and percentages. Qualitative and categorical variables consist of: marital status (single/married/divorced), marital relationship (stable/conflicted/not assessed), current position (manager/staff), number of children (0, 1, ≥ 2), night shift (yes/no), number of duties per month (0, 1–7, ≥ 7), sleep disorders, adequate salary (yes/no/don’t know/not evaluated), overload work (yes/occasionally/no), overtime work (yes/no), job satisfaction (yes/no/partly satisfied/acceptable), level classification of EE, DP, and PA (low/average/high) in MBI–HSS assessment, and FSFI questionnaires (yes/no). Sleep disorders are conditions that affect the quality, amount, and timing of sleep at night.^28^

The Cronbach function of the multilevel package was used to obtain the Cronbach alpha, suggesting good internal consistency. We used independent the *t*-test and one-way ANOVA test for testing the mean difference. Using the correlation coefficient (r) to reveal the association between sexual disorders and burnout depended on the distribution of data. Statistical significance was set at a *p* value <0.05.

## Results

A total of 485 nurses and midwives working at our hospital were eligible for inclusion criteria of burnout assessment. In the overall study population, Table 1 shows the respondents aged from 30 to 50 years old, accounting for 65.5%. In addition, 82.7% of female participants lived with their partner and had at least one child (86.6%). The rest of the study population was still single and had no children. More than half of female nurses and midwives (68.2%) participated in the night shift and 52.6% had more than 7 duties per month. Years of service lasting 10–30 years occupied up to 78.7%.

**Table 1.** Baseline Characteristics of the Study Population

Characteristics	*N* = 485	Percentage (%)
Age group		
22 to <31	85	17.5
31 to <40	152	31.3
40 to <50	166	34.2
50 to 55	82	16.9
Mean age (years)	39.6 ± 8.4	
Marital status		
Single	54	11.1
Married/living with partner	401	82.7
Divorced/widowed	30	6.2
Marital relationship		
Stable	393	5.2
Conflicted	18	85.6
Refused to answer/prefers to not answer	74	9.3
Current position		
Manager	30	6.2
Staff	455	93.8
Number of children (%)		
0	65	13.4
1	124	25.6
≥2	296	61.0
Duration of work (years)		
<10	109	22.5
10 to <20	177	36.5
20 to <30	156	42.2
≥30	43	8.8
Mean duration (X ± SD)	16.6 ± 8.4	
Night shift		
Yes	331	68.2
No	154	31.8
Number of duties/ per month^a^		
0^b^	147	30.3
1–7	83	17.1
≥7	255	52.6
Sleep disorders		
Yes	209	43.1
No	276	56.9
Monthly income (million VND)		
<10	25	5.2
10 to <20	415	85.6
≥20	45	9.3
Salary is enough for current work/adequate salary		
Yes	406	83.7
No	69	14.2
Don’t know/Not evaluated	10	2.1
Overload work		
Yes	116	23.9
Occasionally	297	61.2
No	72	14.8
Overtime work		
Yes	114	23.5
No	371	76.5
Job satisfaction		
Yes	455	93.8
No	22	4.5
Partly satisfied/acceptable	8	1.7

^a^ Duties include night shift or day shift.

^b^ At Tu Du Hospital, the nurses and midwives had no duties if they worked in some units such as Examination Room Department, Fertility Department, and Family Planning Department. In addition, the nurses and midwives play a role of manager. Some other special cases such as the nurses and midwives with children less than 6 months and the nurse/midwives with severe-moderate medical diseases were exempted from the duties.

In terms of sexual activity, 426 female health care workers had at least one sexual intercourse within 1 month. Among them, 42.0% of participants had decreased desire (179/426 cases), other disorders such as decreased excitement (13.1%), insufficient lubrication (12.2%), feeling difficulty in achieving orgasm (13.4%), dissatisfaction (12.0%), and pain during intercourse (13.6%) were observed. The overall prevalence of sexual dysfunction was 12.0% (51/426 cases) and the mean score was 59.6 ± 21.0 (Table 2).

**Table 2.** Sexual Dysfunction Among the Study Population

Characteristics	*N* = 426	Percentage (%)
Decreased sexual desire		
Yes	179	42.0
No	247	58.0
Mean score	4.7 ± 1.6	
Decreased arousal		
Yes	56	13.1
No	370	86.9
Mean score	10.3 ± 4.2	
Insufficient lubrication		
Yes	52	12.2
No	374	87.8
Mean score	14.0 ± 5.9	
Feeling difficult to achieve orgasm/anorgasmia		
Yes	57	13.4
No	369	86.6
Mean score	10.2 ± 3.5	
Dissatisfaction		
Yes	51	12.0
No	375	88.0
Mean score	10.0 ± 3.4	
Dyspareunia/painful intercourse		
No	58	13.6
Yes	368	86.4
Mean score	10.5 ± 4.5	
Overall sexual disorders		
Yes	51	12.0
No	375	88.0
Mean score	59.6 ± 21.0	

With regard to subscales of the burnout syndrome, a high level of EE, DP, and PA were recorded at 13.2%, 6.0%, and 18.4% of the study participants, respectively. If the participant was evaluated to achieve at least one of three subgroups of EE, DP, or diminished PA at a high level, this participant was considered to have burnout syndrome. As a result, the burnout syndrome was noted at 27.0% (131/485 cases) in our study (Table 3).

**Table 3.** Burnout Syndrome in the Study Population

Features	*N* = 485	Percentage %
Emotional exhaustion (EE)		
Low	352	72.6
Average	69	14.2
High	64	13.2
Mean score	15.7 ± 9.3	
Depersonalization (DP)		
Low	410	84.5
Average	46	9.5
High	29	6.0
Mean score	2.8 ± 3.9	
Personal achievement (PA)		
Low	325	67.0
Average	71	14.6
High	89	18.4
Mean score	40.5 ± 8.8	
Overall burnout syndrome		
Yes	131	27.0
No	354	73.0

To better understand burnout syndrome, the study highlights the factors affecting EE and PA among participants. Young age, duration of work years <10 years, staff position, low monthly income, sleep disorders, overtime work, overload work, and job unsatisfaction were significantly related to high EE score. Regarding PA scores, the study found that sleep disorders, overtime work, overload work, and job dissatisfaction were more likely related to high PA scores (Table 4).

**Table 4.** Association Between Emotional Exhaustion, Personal Achievement, and Sociodemographic, Job Characteristics

Characteristics	EE score		PA score	
	Mean	*p* value	Mean	*p* value
Age group				
20 to <31	18.3	<0.01^a^	40.1	0.10^a^
31 to <40	17.1		41.0	
40 to <50	13.7		39.3	
50to 55	14.3		42.1	
Mean age	r = −0.2	<0.01^c^	r = 0.04	0.43^c^
Marital status				
Single	15.8	0.47^a^	39.4	0.26^a^
Married/cohabitation	15.8		40.3	
Divorced/widowed	13.6		42.9	
Marital relationship				
Stable	15.7	0.74^a^	40.6	0.52^a^
Conflicted	17.2		38.2	
Not assessed	15.3		40.3	
Number of children				
0	16.9	0.70^a^	39.5	0.46^a^
1	15.6		41.1	
≥2	15.4		40.4	
Duration of worked years (years)				
<10	18.0	<0.001^a^	40.1	0.88^a^
10 to <20	16.3		40.3	
20 to <30	13.6		40.6	
≥30	14.7		41.2	
Mean duration of worked years	r = −0.2	<0.001^c^	r = 0.1	0.26^c^
High-level position of job				
Yes	11.1	0.006^a^	43.7	0.03^a^
No	16.0		40.2	
Night shifts				
Yes	15.7	0.90^b^	40.2	0.31^b^
No	15.6		41.0	
Sleep disorders				
Yes	19.2	<0.001^a^	37.8	<0.001^a^
No	12.5		42.5	
Monthly income (million VND)				
<10	23.9	<0.001^a^	40.0	0.21^a^
10 to <20	15.5		40.0	
≥20	12.6		42.6	
Overtime work				
Appropriate	14.1	<0.001^a^	41.0	<0.003^a^
Inappropriate	23.7		37.7	
Work overload				
Yes	23.4		36.2	
Occasionally	13.7	<0.001^a^	41.5	<0.001^a^
No	11.4		43.1	
Overtime work				
Yes	15.8	0.99^b^	40.5	0.95^b^
No	15.6		40.4	
Job satisfaction				
Yes	14.7	<0.001^a^	40.9	<0.001^a^
No	29.8		32.9	

^a^ One-way ANOVA.

^b^ Independent *t*-test.

^c^ Pearson correlation.

Table 5 shows that EE at work is related to overall sexual dysfunction and almost all sexual dysfunction domains except lubrication with a weak negative correlation coefficient (r variable from −0.10 to −0.13). Contrariwise, DP was not associated with sexual dysfunctions. Decreased PA was related with overall sexual dysfunction, anorgasmia, and dyspareunia with a weak positive correlation coefficient (r variable from 0.10 to 0.14).

**Table 5.** Correlation Between Burnout Syndrome and Sexual Dysfunctions

Sexual disorders burnout	Desire	Arousal	Lubrication	Orgasm	Satisfaction	Pain	Total
EE	*p* = 0.02 r = −0.12	*p* = 0.02 r = −0.11	*p* = 0.06 r = −0.09	*p* = 0.01 r = −0.13	*p* = 0.02 r = −0.11	*p* = 0.03 r = −0.10	*p* = 0.02 r = −0.12
DP	*p* = 0.08 r = −0.08	*p* = 0.17 r = −0.07	*p* = 0.12 r = −0.08	*p* = 0.07 r = −0.09	*p* = 0.21 r = −0.06	*p* = 0.17 r = −0.08	*p* = 0.10 r = −0.08
PA	*p* = 0.76 r = 0.02	*p* = 0.15 r = 0.07	*p* = 0.07 r = 0.09	*p* = 0.01 r = 0.14	*p* = 0.08 r = 0.09	*p* = 0.04 r = 0.10	*p* = 0.04 r = 0.10

“r” was calculated from Pearson correlation.

## Discussion

Currently, several tools have been applied to evaluate burnout such as MBI, Professional Quality of Life, Oldenburg Burnout Inventory, Copenhagen Burnout Inventory, Stanford Professional Fulfillment Index, and Mini-Z Survey.^29^ Nevertheless, the MBI–HSS remains a reliable and factorially valid instrument to evaluate burnout syndrome in health professionals.^2,30,31^ Our study underlines the prevalence of burnout syndrome among female nurses and midwives was 27.0%. In general, our burnout prevalence is a moderate figure compared to the prevalence in other previous studies worldwide (33.3%−57.4%).^8,32,33^ Across the world, the variable prevalence of burnout depends on population characteristics, work environment as well as the policy of the hospital, society, and the government.^34^ Particularly, our study was conducted after the COVID-19 pandemic, and the prevalence was commonly higher during the COVID-19 pandemic.^11,35^

With respect to subscales of burn-out syndrome, a high level of EE, DP, and PA were recorded at 13.2%, 6.0%, and 18.4% of the study participants, respectively. In accordance with Albendín-García, the systematic-review study revealed the prevalence of a high level of EE ranging from 9.3% to 38.6%, a high-level of DP ranging from 3.8% to 14.5%, and a high-level of PA reduction ranged from 6.7% to 58.0%.^36^ Similarly, Mollart et al. found that almost two-thirds (60.7%) of midwives experienced moderate to high levels of EE, a third (30.3%) scored low PA, and a third (30.3%) experienced DP related to burnout.^37^ In Indonesia, Putra et al. revealed a high level of burnout was recognized in 34.8% of the respondents in the area of EE, 24.3% in the area of DP, and 24.5% in the area of reduced PA.^38^

Midwives who had spent longer in the profession scored low burnout levels.^37^ In our present study, some factors were significantly found to be linked with high EE scores such as younger age less than 40 years old, year of experience less than 10 years, staff position, low monthly income, sleep disorders, overtime work, overload work, and job dissatisfaction. The study also demonstrated that sleep disorders, overtime work, overload work, and job dissatisfaction were more likely related to high PA scores. In line with the findings of Putra et al., marital status, work experience, and employment status were factors related to burnout.^38^ Oe et al. also demonstrated that marital status in Japan influenced PA.^39^ Most recently, Andina-Diaz et al. have conducted a systematic review based on 36 studies with a total of 17,364 participants. There were higher levels of burnout in midwives who were single, under 35–40 years of age, with less than 10 years of experience, and those with young children. Stress, anxiety, and depression, as well as the emotional impact of traumatic events, have been described as related psychological factors.^40^ In Vietnam, among 147 health care professionals from various medical establishments, Nguyen et al. demonstrated that resilience is an important factor in the association between stress and burnout. In addition, gender impacts the interplay between stress and mental resilience. Particularly, the study of Nguyen et al. revealed that inequalities in stress, burnout, resilience, and social support relating to demographic and professional factors.^17^

Night shift increases the potential risk of sleep disorders among health care workers. The results obtained in the present study show that the group with night shift developed seriously burnout syndrome compared with the group without night shift. Concisely, fixed and rotating night shifts seemed to be associated with a high rate of “burnout” at high levels, with the highest scores recorded for self-doubt and low personal achievement.^41^ Moreover, the night shift was associated with the prevalence of sexual dysfunction in health care workers. A study of Polish midwives found that shift work negatively affects sexual and reproductive health. Appropriate management is required to minimize shift rotation and implement work schedules that allow female health care workers to recover and maintain adequate family and social life.^42^ In line with Papaefstathiou et al. in Greece, job stress decreased lubrication and orgasm; thus, these authors suggested that occupational stress affects female sexual problems.^31^

The prevalence of sexual disorders was 12.0% in our study. The most common category of disorders is decreased desire, accounting for nearly 42.0%. Our sexual disorders prevalence is lower than that of Farah’s study conducted on 1400 female staff from a level-III hospital in Singapore using the FSFI scale; the finding showed that the rate of female staff with sexual dysfunction was up to 56.0%.^43^ In Malaysia, Grewal et al. revealed that female sexual desire disorder is common among female health care personnel, affecting nearly one in five women (18.9%). Women with low sexual desire were more likely to have higher educational attainment (OR = 3.06; 95% CI: 1.22–7.66), lower frequency of sexual intercourse (OR = 12.81; 95% CI: 4.43–37.83), two or more children (OR = 3.05; 95% CI: 1.02–9.09), duration of marriage of 20 years or more (OR = 2.62; 95% CI: 1.27–5.40), and a spouse with erectile dysfunction (OR = 2.86; 95% CI; 1.08–7.56).^44^ Particularly, sexual dysfunction was detected in 100% of the nurses (76/76 cases) in the study of Utkualp et al.^45^ Recently, Tan et al. have also addressed some risk factors in association with sexual dysfunction including older age, dissatisfaction with income, a poor physician–patient relationship, and poor sleep in primary hospitals.^15^

Among participants with burnout syndrome, work-related EE was significantly associated with general sexual status except lubrication. The negative correlation coefficient with (r) ranged from −0.10 to −0.13 shows that the higher the EE score (severe burnout), the lower the FSFI score (more severe sexual disorders). Decreased PA was associated with overall sexual status, anorgasmia, and dyspareunia with the correlation coefficients (r) of 0.10, 0.14, and 0.10 showing that the higher the PA score (no burnout), the higher the FSFI score (no sexual disorders). Both of these arguments demonstrate a positive correlation between burnout and sexual dysfunction in female nurses and midwives. However, the correlation coefficient remains weak in our findings. Our data are somewhat similar to that of Tan et al., who showed a significant positive correlation between DP and sexual disorders drive (*r* = 0.508, *p* < 0.001), sexual arousal (*r* = 0.521, *p* < 0.001), lubrication (*r* = 0.432, *p* < 0.001), orgasm/erection (*r* = 0.420, *p* < 0.001), and sexual satisfaction (*r* = 0.434, *p* < 0.001).^15^

### Strengths and limitations

Aside from burnout syndrome, sexual disorders are less likely documented among female health care workers. This barrier is similar to other countries in Asia. Interestingly, this is a novel study evaluating the association between burnout and sexual disorders in female nurses and midwives in Vietnam, where the sexual issues are commonly difficult to share. Previously, other studies mentioned only the burnout among Vietnamese nurses and midwives.^46,47^ The study was conducted at a tertiary referral hospital in the south of Vietnam; thus, the initial findings with a large sample size are important. The response rate and completion of the survey were considerably high in our study. The study used the Google Online Form and created a comfortable environment for all participants; thus, the responses could be accurate. Moreover, the survey form was distributed to 24 units in our hospital, so results could be representative of the overall female nurses and midwives.

Likewise, the study should be interpreted in the context of several potential limitations. First, the study was exclusively conducted at a single center and focused on the health care personnel working in the obstetrics and gynecology field. Furthermore, a cross-sectional design was limited to determine the causal conclusions. Similar to other survey-based studies, measurement errors due to recall bias and social desirability bias must be taken into consideration. Since this study used a survey form with qualitative and categorical variables, the responses may be individually subjective and depending on the perception of participants, without concise evidence. In this study, we analyzed the relationship between burnout and sexual disorders but did not evaluate or adjust for other factors that are known to affect sexual function. The study investigated the sociodemographic and job characteristics but did not assess lifestyle factors such as exercise, smoking, alcohol intake, and obesity as well as physical health. However, the consumption of alcohol and tobacco among Vietnamese women is probably low. It is necessary to conduct multicentric studies to get more consistent results. A multivariate logistic regression model ought to be developed to calculate the risk of burnout and sexual disorders. At the time of the study, the research team had not yet found a proper intervention for the participants explored with burnout syndrome and sexual disorders.

## Conclusion

In summary, the burnout prevalence among female nurses and midwives working at Tu Du Hospital was 27.0%, and work-related factors were definitely the main factors associated with burnout syndrome. In addition, the prevalence of sexual disorders in female nurses and midwives was 12.0%. EE and reduced PA were related to sexual disorders in female nurses and midwives. This new insight may aid in developing decision-making strategies and social support to mitigate burnout and promote professional fulfillment for female nurses and midwives.

## Data Sharing Statement

The datasets generated and/or analyzed during the current study are not publicly available due the restricted policy of the hospital but are available from the corresponding author on reasonable request.

## Abbreviations Used

DP: depersonalization
EE: emotional exhaustion
FSFI: Female Sexual Function Index
MBI-HSS: Maslach Burnout Inventory–Human Services Survey
PA: personal accomplishment
