# Supplementary material for: Burnout Syndrome and Sexual Disorders Among Vietnamese Female Nurses and Midwives at Tu Du Hospital: A Frontline Hospital-Based Cross-Sectional Study
Source: Womens Health Rep (New Rochelle). 2025 Jul 2;6(1):660–70. doi: 10.1089/whr.2024.0193 (PMC12241844; doi:10.1089/whr.2024.0193)
Supplement: Supplementary Table S1 [file whr.2024.0193_supplementary_table_s1.docx]

**Supplemental Table 1. The number of female nurses and midwives accepted to be enrolled and eligible for inclusion criteria in each unit at Tu Du Hospital, Vietnam**

| Order | Name of department (unit) | N |
| --- | --- | --- |
| 1 | Emergency Bloc A Dept | 22 |
| 2 | Emergency Bloc B Dept | 18 |
| 3 | Imaging Diagnosis Dept | 20 |
| 4 | Post-operative care Dept | 20 |
| 5 | Infertility Dept | 17 |
| 6 | Reanimation Dept | 23 |
| 7 | Delivery Dept Bloc A | 18 |
| 8 | Delivery Dept Bloc B | 18 |
| 9 | Examination Bloc N Dept | 22 |
| 10 | Family Planning Dept | 21 |
| 11 | Gynecologic Dept | 20 |
| 12 | Laparoscopic Dept | 21 |
| 13 | Examination Bloc M Dept | 20 |
| 14 | Anesthesia Bloc H Dept | 15 |
| 15 | Anesthesia Bloc B Dept | 20 |
| 16 | High-risk Pregnancy Bloc A Dept | 20 |
| 17 | High-risk Pregnancy Bloc B Dept | 20 |
| 18 | Obstetric Bloc H Dept | 21 |
| 19 | Obstetric Bloc M Dept | 20 |
| 20 | Obstetric Bloc N1 Dept | 18 |
| 21 | Obstetric Bloc N2 Dept | 22 |
| 22 | Neonatal Dept 1 | 20 |
| 23 | Neonatal Dept 2 | 15 |
| 24 | Gynecologic Oncology Dept | 20 |
|  | Total | 485 |

Dept: Department
